# High resolution fingerprinting of single and double-stranded RNA using ion-pair reverse-phase chromatography

**DOI:** 10.1016/j.jchromb.2018.11.027

**Published:** 2019-01-01

**Authors:** Alison O. Nwokeoji, Mark E. Earll, Peter M. Kilby, David E. Portwood, Mark J. Dickman

**Affiliations:** a Department of Chemical and Biological Engineering, Mappin Street, University of Sheffield, S1 3JD, UK; b Syngenta, Jealott's Hill International Research Centre, Bracknell, Berkshire RG42 6EY, UK

## Abstract

The emergence of new sustainable approaches for insect management using RNA interference (RNAi) based insecticides has created the demand for high throughput analytical techniques to fully characterise and accurately quantify double stranded RNA (dsRNA) prior to downstream RNAi applications. In this study we have developed a method for the rapid characterisation of single stranded and double stranded RNA using high resolution RNase mapping in conjunction with ion-pair reverse-phase chromatography utilising a column with superficially porous particles. The high resolution oligoribonucleotide map provides an important ‘fingerprint’ for identity testing and bioprocess monitoring. Reproducible RNA mapping chromatograms were generated from replicate analyses. Moreover, this approach was used to provide a method to rapidly distinguish different RNA sequences of the same size, based on differences in the resulting chromatograms. Principal components analysis of the high resolution RNA mapping data enabled us to rapidly compare multiple HPLC chromatograms and distinguish two dsRNA sequences of different size which share 72% sequence homology. We used the high resolution RNase mapping method to rapidly fingerprint biomanufactured dsRNA across a number of different batches. The resulting chromatograms in conjunction with principal components analysis demonstrated high similarity in the dsRNA produced across the different batches highlighting the potential ability of this method to provide information for batch release in a high throughput manner.

## Highlights

• Developed high resolution RNase mapping in conjunction with IP RP HPLC
• Provides an important ‘fingerprint’ for identity testing and bioprocess monitoring
• Rapidly distinguishes different RNA sequences of the same size
• Method used to rapidly fingerprint biomanufactured dsRNA across a number of different batches

## 1 Introduction

With the emergence of suitable large scale production systems for the biomanufacturing of double stranded RNA (dsRNA) for RNAi applications, the development of high throughput methods that facilitate the rapid characterisation of dsRNA is required. Mass spectrometry based methods offer a powerful approach to characterise RNA. RNase mass mapping methods have been developed and used for the identification and quantification of RNA and RNA post transcriptional modifications [[1], [2], [3], [4], [5], [6], [7], [8], [9], [10]]. Typical workflows involve the purification of the RNA [[11], [12], [13], [14]], prior to RNase digestion into smaller oligoribonucleotides that are more amenable for chromatographic separation and intact mass measurements [[15], [16], [17], [18]]. Additional sequence information from the oligoribonucleotides can be obtained using tandem mass spectrometry (MS/MS) [19,20]. However, the analysis of nucleic acids in particular RNA, *via* mass spectrometry has proved more difficult compared to the routine applications of proteomics to study biological systems. This is due to the difficulty in purifying and enriching biological RNA, the formation of RNA-metal ion adducts and problems associated with sequencing RNAs due to poor fragmentation in tandem mass spectrometry experiments. Furthermore, there is traditionally a trade-off between chromatographic performance and mobile phase conditions necessary to obtain high sensitivity during electrospray mass spectrometry analysis.

Recent approaches have further developed the use of RNase mass mapping for the characterisation of dsRNA [21]. RNase digestion of the dsRNA was performed, using both RNase A and a novel method utilising RNase T1 for RNase mass mapping approaches to further characterise the dsRNA using liquid chromatography interfaced with mass spectrometry. However detailed analysis of RNA using mass spectrometry is time consuming, requires significant technical expertise and expensive instrumentation. In addition, limited software exists for the automated analysis of typical mass spectrometry data from RNase mass mapping approaches.

In the production of biopharmaceutical proteins peptide mapping with reversed-phase (RP) chromatography has been used for the analyses of recombinant protein biopharmaceuticals delivering comprehensive characterisation of products [[22], [23], [24], [25], [26]]. In addition, these methods are also employed for subsequent lot-to-lot identity testing (‘fingerprinting’) in support of GMP production. When interfaced with mass spectrometry, the method enables the identification of proteins and their variants, characterisation of post-translational modifications (PTMs), and confirmation of protein sequences. More recently, Ultra High Performance Liquid Chromatography (UHPLC) has been employed, demonstrating superior resolution, higher sensitivity, and much shorter analysis times compared to traditional HPLC approaches. Peptide mapping approaches have been utilised to assess the stability of biopharmaceuticals in conjunction with the detection of amino acid oxidation and deamidation [27]. Furthermore, these approaches provide information regarding quality control, analysis of batch-to-batch consistency and the stability of biopharmaceuticals [24,28,29].

In this study we have developed a method for the rapid characterisation of single stranded and double stranded RNA using high resolution RNase mapping in conjunction with ion-pair reverse-phase chromatography utilising superficially porous particles. This mapping approach can be applied to RNA in a similar way that peptide mapping is used in the production of proteins. Principal components analysis was employed to analyse the repeatability of replicate analysis, detect minor differences between different RNA and analyse batch-to-batch variability in the production of ssRNA and dsRNA.

## 2 Materials and methods

### 2.1 Chemicals and reagents

Genes were synthesised by GeneArt® Gene Synthesis (Invitrogen Life Technologies). Ampicillin sodium salt, tetracycline hydrochloride, isopropyl β‑d‑1‑thiogalactopyranoside (IPTG) ≥99%, sodium dodecyl sulphate (SDS) sodium chloride (NaCl), dimethyl sulfoxide (DMSO) were from Sigma Aldrich.

### 2.2 Expression and purification of dsRNA using *E. coli* HT115 (DE3)

The *E. coli* strain, HT115 (DE3) [30] was obtained from Cold Spring Harbor Laboratory, NY, USA. Plasmid pDome11 contained an in-house designed 481 bp sequence flanked on both sides with T7 promoters was transformed into *E. coli* HT115 (DE3) cells. The pDome11 transformed cells were grown in culture and induced with IPTG to express dsRNAs as previously described [21].

RNA purification was performed using the RNASwift method as previously described [31] with minor modifications. In brief, a pellet of 10^9^ *E. coli* cells was re-suspended in 200 μL of pre-warmed lysis buffer (50% DMSO, 0.1% SDS, 0.5 M NaCl, 70 °C) and incubated at 70 °C for 3 min followed by 3 minute incubation at 37 °C. 200 μL warm solution (4% SDS, 0.5 M NaCl, 70 °C) was added followed by addition of 100 μL 5 M NaCl. This was centrifuged at 20,238*g* for 4 min and supernatant transferred to a new Eppendorf tube. 100 μL 60% isopropanol was added and purified using solid phase extraction (SPE) as essentially described [31]. It is important to ensure the same extraction and purification procedure is used for reproducible IP-RP-HPLC chromatograms following RNase digestion.

RNA concentrations were determined using a NanoDrop™ 2000c spectrophotometer (ThermoFisher Scientific) by absorbance at 260 nm normalized to a 1.0 cm (10.0 mm) path and A_260/280_ and A_260/230_ ratios obtained [32]. Additional analysis of the RNA was performed using ion-pair reverse phase chromatography.

### 2.3 Ion-Pair Reverse Phase High Performance Liquid Chromatography (IP-RP-HPLC)

Samples were analysed by IP-RP-HPLC on a passivated Agilent 1100 series HPLC using a Proswift RP-1S Monolith column (50 mm × 4.6 mm I.D. ThermoFisher). Chromatograms were generated using UV detection at a wavelength of 260 nm. The chromatographic analysis was performed using the following conditions: Buffer A 0.1 M triethylammonium acetate (TEAA) pH 7.0 (Fluka, UK); Buffer B 0.1 M TEAA, pH 7.0 containing 25% acetonitrile (ThermoFisher). RNA was analysed using the following gradient. Gradient starting at 22% buffer B to 27% in 2 min, followed by a linear extension to 62% buffer B over 15 min, then extended to 73% buffer B over 2.5 min at a flow rate of 1.0 mL/min at 50 °C.

### 2.4 *In vitro* transcription (IVT) of dsRNA and ssRNA

For dsRNA synthesis *via in vitro* transcription, DNA template was amplified using PCR from the plasmid pCOIV that contains a 686 bp sequence flanked on both sides with T7 promoter sequences and optimised synthetic T7 terminator sequences. In addition, a 561 bp DNA template flanked on both sides by T7 promoters was generated by PCR from within the 686 bp sequence of the pCOIV plasmid. For *in vitro* synthesis of ssRNA, a DNA template was amplified using PCR from the plasmid pCsm40 that contains 521 bp sequence with a T7 promoter. To create a complementary ssRNA, a 521 bp DNA template identical to the first template was used with a T7 promoter on the opposite strand. The following conditions were used: 0.02 U/μL Q5 High-Fidelity DNA Polymerase, 200 μM dNTPs, 0.5 μM each of forward and reverse primer and 10 ng DNA template in a final volume of 50 μL. The following PCR parameters were used: the initial denaturation was 1 cycle of 30s at 98 °C, 30 cycles of 30 s at 98 °C, 30 s at 68 °C, and 30 s at 72 °C and a final extension at 72 °C for 2 mins. dsRNA and ssRNA were then generated using *in vitro* transcription in conjunction with HiScribe™ T7 High Yield RNA Synthesis Kit (New England Biolabs): 10 mM NTPs, 1× reaction buffer, 1 μg DNA template and 2 μL HiScribe™ T7 polymerase in 20 μL RNase-free water. Compositions of the dsRNA and ssRNAs are shown in the Supplementary Table 1.

### 2.5 RNase LC-UV mapping

Following purification, 2.5 μg of dsRNA in RNase-free water was incubated with 100 ng RNase A at 37 °C for 30 min in a volume of 10 μL. For RNase T1 mapping, 2.5 μg of purified dsRNA in 50% DMSO was incubated at 90 °C for 30 s and allowed to cool to room temp. 2000 U RNase T1 was added and reaction mix incubated for 15 min at 37 °C in a volume of 10 μL. Subsequently, 2.5 μg of digested dsRNA was analysed using IP-RP-HPLC-UV using an Accucore C18 column (2.6 μm superficially porous silica particles 80 Å pore size 150 mm × 2.1 mm ID). LC buffer A: 80 mM 1,1,1,3,3,3‑Hexafluoro‑2‑propanol (HFIP, Sigma-Aldrich) with 20 mM TEAA (Sigma-Aldrich), LC buffer B: LC buffer A with 50% acetonitrile (v/v) (ThermoFisher). Starting with 6% buffer B, an isocratic step was performed for 2 mins followed by a linear extension to 20% B in 23 min, then extension to 30% B over 3 min. Extend to 50% B for 1 min and subsequently to 6% B in 0.5 min and hold at 6% B for 3 min at a flow rate of 300 μL min^−1^, using UV detection at a wavelength of 260 nm. All RNase mapping IP-RP-HPLC analyses were performed on a U3000 RSLC system (Thermo Scientific). Data analysis was performed in Chromeleon Software v7.2 (Thermo Scientific). Average Relative Standard Deviation (%RSD) were calculated for both retention time and peak area using Chromeleon using the individual RSD for selected peaks (>5 mAU) in the chromatogram.

### 2.6 Principal components analysis

The Principal Components Analysis was carried out using SIMCA-P 14.0 Software (Sartorius Stedim Data Analytics). The data were exported from Chromeleon 7.0 (ThermoFisher) as Excel files. The data were reduced by binning into 10 datapoints width bins using the prospectr package in the R-language for statistical computing. This left at least 19 points across the narrowest chromatographic peak but reduced the size of the dataset to a more manageable size [33].

## 3 Results and discussion

### 3.1 High resolution RNA fingerprinting using IP-RP-HPLC

**Fig. 1:**
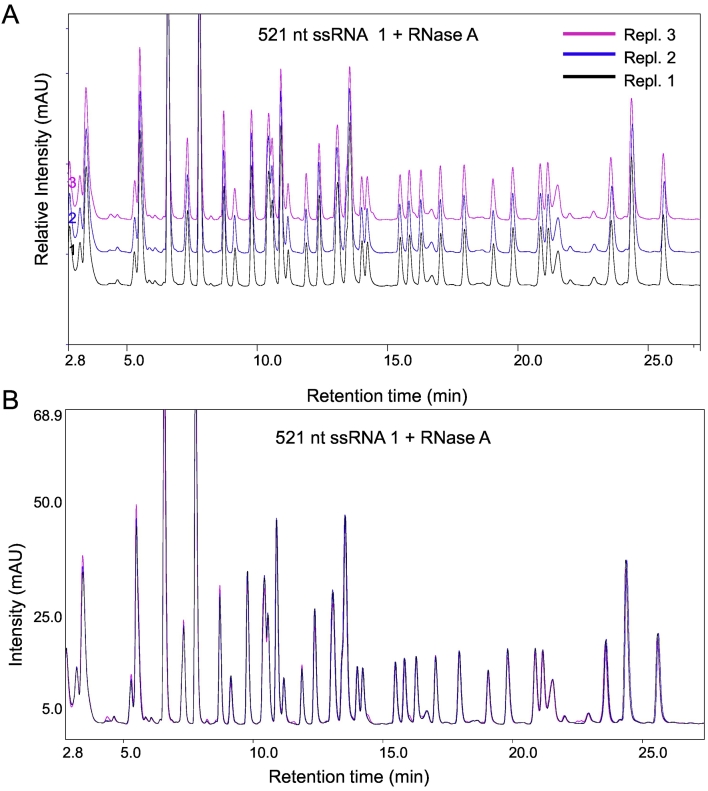
RNase mapping of ssRNA in conjunction with IP-RP-HPLC. A) IP-RP-HPLC chromatogram of 521 nt ssRNA 1 digested with RNase A. Replicate injections of the same sample made on the same day are shown. B) Overlay of the replicate chromatograms from the RNase A digest of the 521 nt ssRNA 1.

A 521 nt ssRNA was generated using *in vitro* transcription and subsequently purified to remove proteins and contaminants from *in vitro* transcription reagents. IP-RP-HPLC analysis in conjunction with a monolithic polystyrene‑divinylbenzene column was used to demonstrate the purity of the ssRNA samples used in this study (see Supplementary Fig. 1A). Subsequently the purified ssRNA was digested with RNase A prior to IP-RP-HPLC analysis in conjunction with IP-RP-HPLC using superficially porous silica particles to generate a high resolution fingerprint of the RNA. We have previously demonstrated the application of superficially porous silica particles for the analysis of nucleic acids [34]. The purified ssRNA was digested with RNase A prior to IP-RP-HPLC analysis (see Fig. 1A). IP-RP-HPLC analysis was performed using 20 mM TEAA, 80 mM HFIP and oligoribonucleotides eluted using an acetonitrile gradient. The results demonstrate the reproducibility of the IP-RP-HPLC method, an overlay of 3 replicate injections of the same sample is shown in Fig. 1B. Average RSD for retention time and peak area for the peaks shown in the chromatogram were determined as 0.07% and 4.8% respectively. These results demonstrate the ability to rapidly generate a reproducible chromatographic profile of the digested ssRNA using high resolution IP-RP-HPLC.

**Fig. 2:**
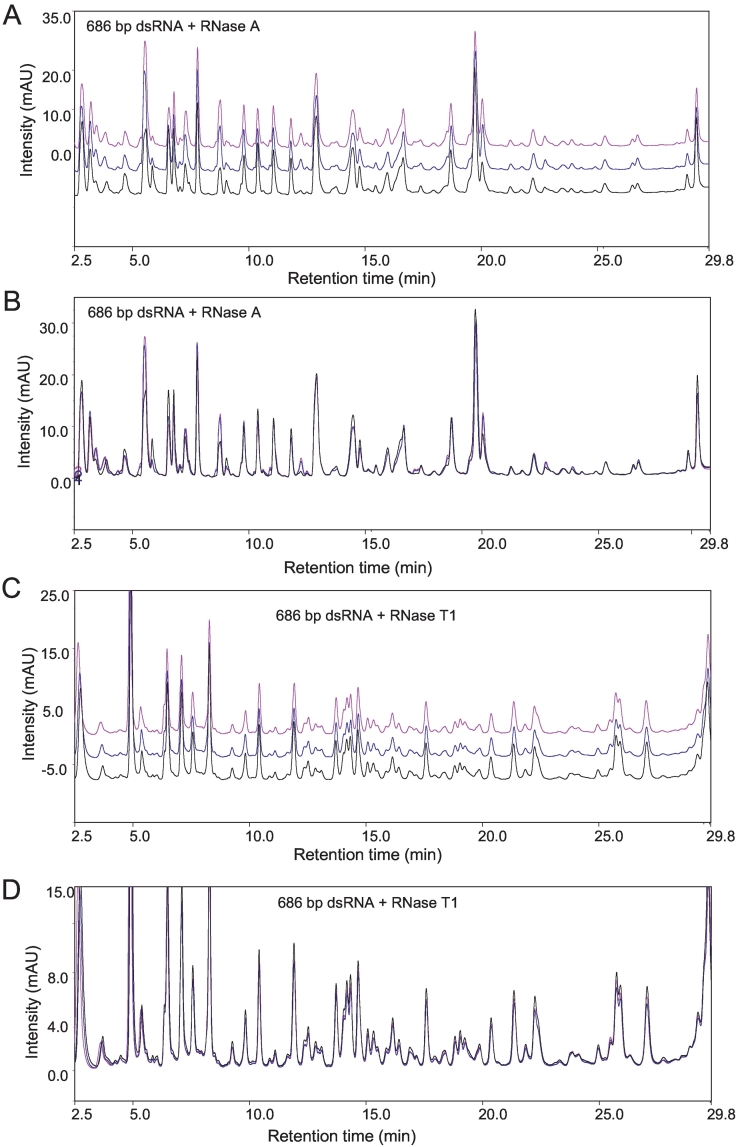
High resolution RNase mapping of dsRNA in conjunction with IP-RP-HPLC A) IP-RP-HPLC chromatogram of 686 bp dsRNA digested with RNase A. Replicate injections of the same sample made on the same day are shown. B) Overlay of the replicate chromatograms from one RNase A digest of the 686 bp dsRNA. C) IP-RP-HPLC chromatogram of 686 bp dsRNA digested with RNase T1. Replicate injections of the same sample made on the same day shown. D) Overlay of the replicate chromatograms from one RNase T1 digest of the 686 bp dsRNA.

To further validate the method, the analysis was applied to dsRNA. dsRNA provides a more complex sample, as typically twice as many oligoribonucleotides are generated compared to ssRNA following RNase digestion. This therefore presents a more challenging sample for IP-RP-HPLC analysis. A 686 bp dsRNA was synthesised using *in vitro* transcription and purified prior to analysis by IP-RP-HPLC to confirm the purity of the dsRNA used in this study (see Supplementary Fig. 1C). The purified dsRNA was subsequently digested with RNase A, which cleaves each strand of dsRNA 3′- to C or U bases, and analysed using IP-RP-HPLC analysis (see Fig. 2A/B). The results show the more complex mixture of oligoribonucleotides generated from the 686 bp dsRNA in contrast to the 521 nt ssRNA (see Fig. 1A). These results show that in addition to providing a rapid fingerprint of the ssRNA, such approaches can also provide a reproducible fingerprint of more complex dsRNA. Average RSD for retention time and peak area for the peaks shown in the chromatogram were determined as 0.1% and 13.6% respectively. dsRNA not only generates a more complex mixture of oligoribonucleotides upon RNase A digestion, but presents further challenges for RNase mapping approaches as most RNase are single strand-specific nucleases and therefore not active against dsRNA. We have recently demonstrated that RNase T1 can be used in RNase mass mapping of dsRNA in conjunction with the chemical denaturant DMSO [21]. A short thermal denaturation step in the presence of 50% DMSO denatures the dsRNA and prevents subsequent re-annealing. Addition of RNase T1 selectively cleaves the ssRNA on the 3′-side of G residues. Therefore, to further characterise and fingerprint the dsRNA, RNase T1 mapping was also performed in conjunction with high resolution IP-RP-HPLC (see Fig. 2C/D). Average RSD for retention time and peak area for the peaks shown in the chromatogram were determined as 0.09% and 8.5% respectively.

### 3.2 Rapid detection of different RNA fragments using high resolution RNase fingerprinting

**Fig. 3:**
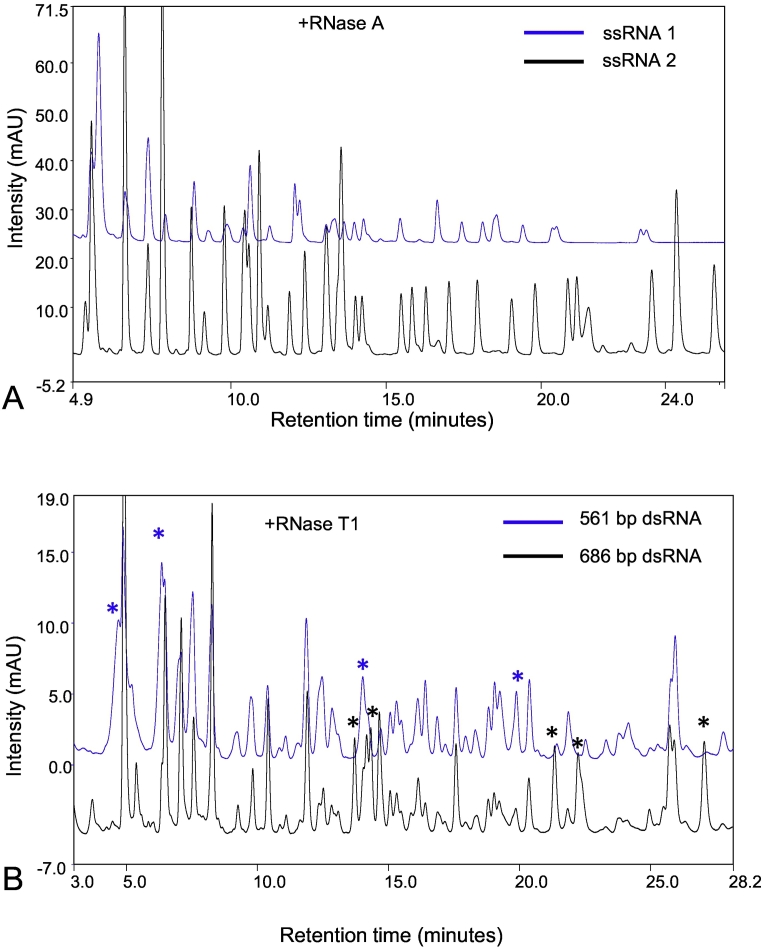
Comparative analysis of different RNA sequences using RNase mapping. A) Overlay of IP-RP-HPLC chromatograms of complementary ssRNA sequences (ssRNA1 and ssRNA2) digested with RNase A. B) Overlay of IP-RP chromatograms of the dsRNA 561 bp and 686 bp digested with RNase T1. Asterisks mark the peaks identified by visual analysis that are different between the chromatograms.

To further demonstrate the ability of the high resolution fingerprinting method to rapidly detect differences between RNA samples, experiments were performed using ssRNAs of the same size but different sequence. The 521 nt ssRNA (1) sequence and its complementary sequence ssRNA (2) were *in vitro* transcribed, purified (see Supplementary Fig. 1A/B) and digested using RNase A prior to IP-RP-HPLC. The resulting chromatograms are shown in Fig. 3A. The results show clear differences in the chromatogram in terms of both retention times and intensity of the oligoribonucleotides generated, enabling us to rapidly distinguish two different ssRNAs (of the same length) based on the chromatogram or fingerprint obtained.

To further investigate the method to rapidly identify different RNAs, we chose to analyse two dsRNAs, a 561 bp and 686 bp dsRNA which share 561 bp of identical sequence. Only 125 bp (18%) of the 686 bp dsRNA is unique in this example. Therefore, only limited numbers of different oligoribonucleotide fragments will be generated either *via* RNase A or RNase T1, making discrimination between these two different RNAs challenging. The 686 and 561 bp dsRNA were *in vitro* transcribed, purified (see Supplementary Fig. 1C/D) and digested using RNase T1 prior to IP-RP-HPLC (see Fig. 3B). The results show as expected many of the oligoribonucleotides generated from each dsRNA are identical, which results in peaks with the same retention time and/or intensity. However, the results also show that a number of peaks could be identified based on differences in either retention time or intensity in the chromatogram (see Fig. 3B marked as asterisks), in contrast to analysis of injection replicates of the same dsRNA (see Fig. 2).

### 3.3 Analysis of lot-to-lot variation in the biomanufacturing of dsRNA using RNase mapping

**Fig. 4:**
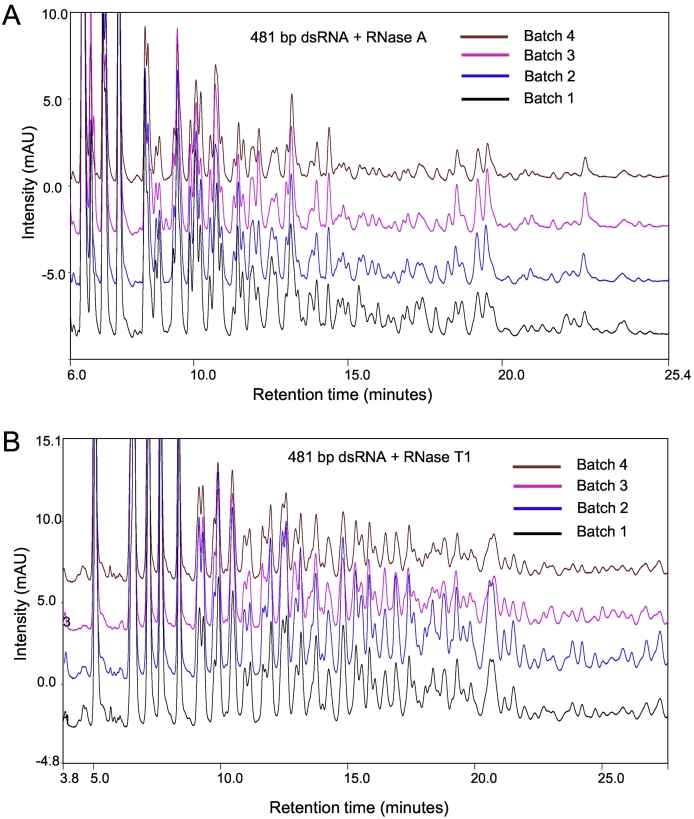
Analysis of batch variability of biomanufactured dsRNA using RNase mapping. IP-RP chromatogram of dsRNA extracted and purified from 4 different batches of *E. coli* cells engineered to express dsRNA. dsRNA digested with A) RNase A or B) RNase T1.

The ability to produce large quantities of dsRNA in either bacterial systems, by *in vitro* transcription, in cell-free systems or *in planta* for RNA interference applications has generated significant demand for the development and application of high throughput analytical tools for analysis of dsRNA. In particular, the development of a method that rapidly characterises dsRNA and assesses potential lot-to-lot variation in the biomanufacturing of dsRNA. The ability to rapidly assess the biomanufactured dsRNA in a high throughput manner without the requirement for specialist mass spectrometers and complex data analysis will provide significant advantages in a biomanufacturing environment. Therefore, in an approach to develop a rapid method for the analysis of biomanufactured dsRNA, 4 separate batches of *E. coli* engineered to express dsRNA were grown and the dsRNA extracted and purified (see Supplementary Fig. 2). The IP-RP-HPLC analysis of the RNase A/T1 digests of the dsRNA is shown in Fig. 4A/B and show that reproducible chromatograms were observed for the dsRNA produced across the 4 different batches in this case. These results demonstrate that in these examples limited lot-to-lot variation of the biomanufactured dsRNA was observed.

### 3.4 Analysis of RNA fingerprinting chromatograms using principal components analysis

Further comparative analysis of the RNAse mapping data was performed using principal components analysis (PCA) to detect patterns of similarities/differences between the resulting chromatograms in an objective manner. PCA is a method of projecting high dimensional data down to simple two dimensional plots that describe the similarity of observations based upon their pattern of variables. The components are projected in order of variance explained so that the first components explain the most prominent features of the data and successive components less important features until eventually all that is left is random variation. Within the SIMCA software the point at which no more useful information is to be extracted is determined by 7 fold validation which is a re-prediction technique. One seventh of the data is left out and re-predicted by the model and the difference between the actual data and the predicted data is expressed as a Q^2^ value which is the cross validated equivalent of R^2^. The point at which this value of predictivity drops is deemed the optimum number of components. This PCA approach has previously been applied to analyse complex chromatographic data [[35], [36], [37], [38], [39]].

**Fig. 5:**
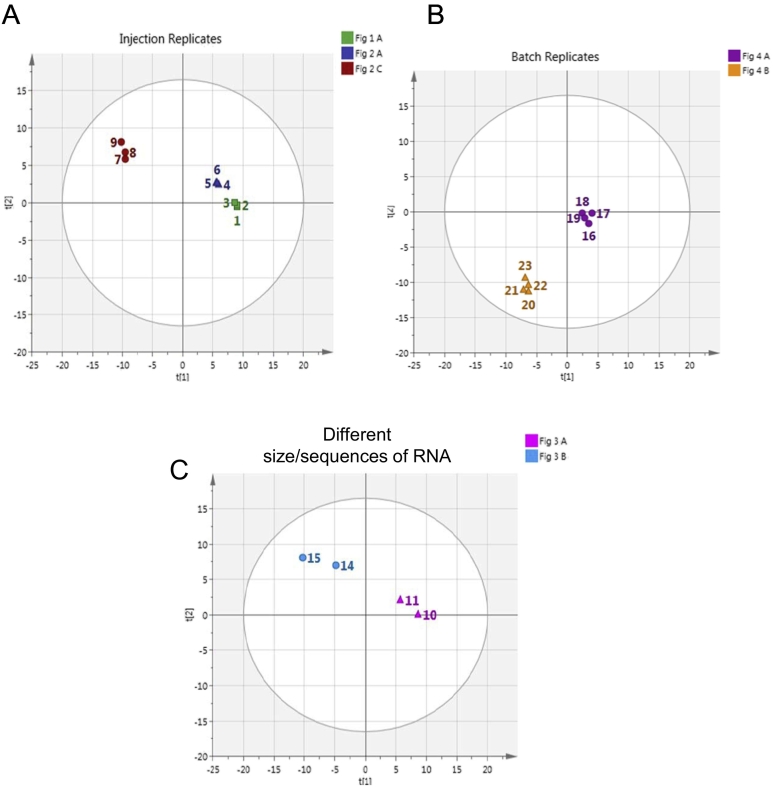
Principal component analysis. A) PCA analysis shows the tight clustering of injection replicates shown in Fig. 1A (1–3 shown in green), Fig. 2A (4–6 shown in blue) and Fig. 2C (7–8 shown in red). B) PCA analysis shows the similarity of the four batches of 481 bp biomanufactured dsRNA digested with either RNase A (16–18 shown in purple) or RNase T1 (20–23 shown in orange). C) PCA analysis shows the difference between the chromatograms of the 521 nt complimentary ssRNAs (10–11 shown in pink) and RNase T1 digest of the 561 bp/686 bp dsRNA that share 72% sequence homology (14–15 shown in blue). Each chromatogram is represented as a point in the plot. The closer together the points the more similar are the chromatograms. Dissimilarity is shown by the distance between the points. (For interpretation of the references to colour in this figure legend, the reader is referred to the web version of this article.)

In this analysis, the data were transformed using Standard Normal Variate (SNV) transform which removes any overall amplitude variation caused by sample dilution or injection variation. The data were also subjected to mean centering and Pareto scaling. The reason for applying Pareto scaling was to up-weight medium peaks without increasing baseline noise as would be the case if unit variance scaling were used. Both transformations were applied within the SIMCA software. The software extracted five principal components by cross validation explaining 89% of the variance but the first two components were sufficient to describe the overall similarity of the chromatograms. The higher order components mainly described minor or artifact features in the chromatograms. The two component Scores plot explains 52% of the variance in the data. Each chromatogram is represented as a point in the plot. The closer together the points the more similar are the chromatograms. Dissimilarity is shown by the distance between the points. For clarity the PCA has been performed on the whole dataset but the result split into three plots which only show the relevant comparisons, namely injection replicates, batch replicates and different size/sequences of RNA (see Fig. 5).

Principal component analysis of the injection replicates of the ssRNA and dsRNA RNase digests is shown in Fig. 5A. The results show the very tight clustering of injection replicates of the RNase A digest of the 521 nt ssRNA (1–3 shown in green), 686 bp dsRNA (4–6 shown in blue) and the RNase T1 digest of the 686 bp dsRNA (7–9 shown in red). In addition, the PCA analysis also shows the similarity of the four batches of biomanufactured 481 bp dsRNA generated from *E. coli* HT115 cells digested using either RNase A (16–18 shown in purple) or RNase T1 digestion (20–23 shown in orange) (see Fig. 5B).

Fig. 5C shows the PCA analysis of the respective chromatograms generated from the RNase digest a 521 nt ssRNA and its complementary sequence (Fig. 3A) and two dsRNAs, a 561 bp and 686 bp dsRNA which share 561 bp of identical sequence (Fig. 3B). The PCA analysis demonstrates the difference between the two ssRNA of the same size but different sequence. In addition, the PCA analysis also highlights the difference in the chromatograms obtained from two dsRNA sequences of different size which share 72% sequence homology (see Fig. 5C).

## 4 Conclusions

In this study we have performed high resolution RNase mapping in conjunction with ion-pair reverse phase chromatography utilising superficially porous particles for the rapid fingerprinting of single stranded and double stranded RNA. The ability to generate a fingerprint based on the IP-RP chromatogram was used to rapidly detect different RNA sequences of the same size, based on differences in the resulting chromatograms. To facilitate the ability to identify small changes in the chromatographic data which cause significant variance among the samples, PCA analysis was performed. Using this approach we were able to distinguish two dsRNA fragments of different size which share 72% sequence identity. Due to the overlapping peaks in these chromatograms, small single nucleotide differences may sometimes be detected. However, it is more likely larger changes in the dsRNA are required before a material could be identified as an incorrect sequence. We used the high resolution RNase fingerprinting method to rapidly analyse dsRNA biomanufactured in *E. coli* across a number of different batches. Moreover the PCA analysis revealed tight clustering of the injection replicates, demonstrating the reproducibility of the chromatography. A cluster of PCA points from a large group of in-specification samples could be used to define the limits of acceptable process variation with any future samples giving analyses outside of this cluster being defined as outside what was expected for that product. The results demonstrate the potential ability to rapidly analyse large numbers of dsRNA, lot-to-lot variability in the production of dsRNA, and detect variations in dsRNA generated for batch release. All without the requirement for interfacing with mass spectrometry and the associated technical expertise required for such analysis.

## Appendix A Supplementary data

Supplementary material Image 1
