# Supplementary material for: High resolution fingerprinting of single and double-stranded RNA using ion-pair reverse-phase chromatography
Source: J Chromatogr B Analyt Technol Biomed Life Sci. 2019 Jan 1;1104:212–9. doi: 10.1016/j.jchromb.2018.11.027 (PMC6329874; doi:10.1016/j.jchromb.2018.11.027)
Supplement: Supplementary file 1 — Supplementary material [file mmc1.pptx]

## Slide 1
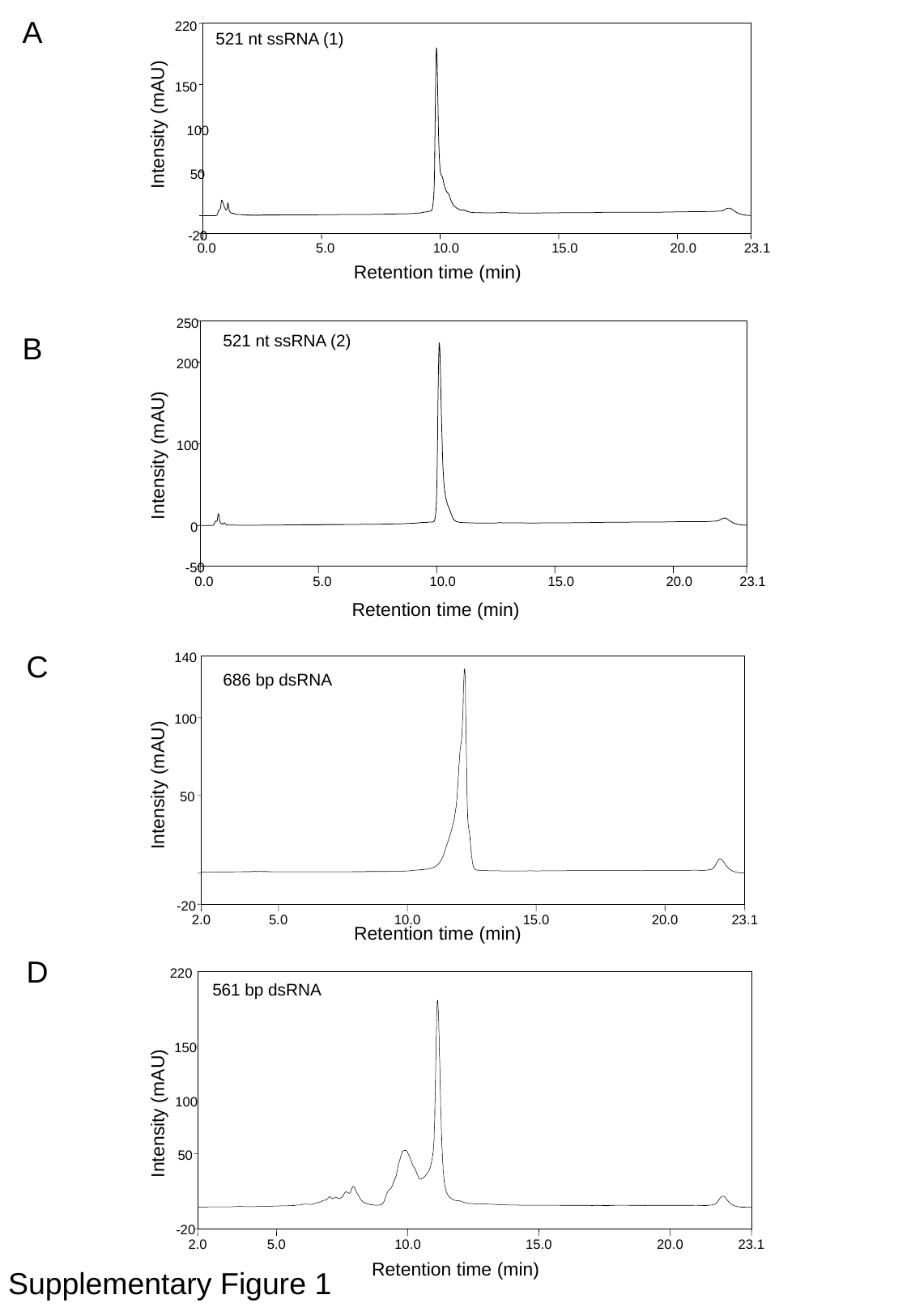

A
220
150
100
50
-20
0.0
5.0
10.0
15.0
20.0
23.1
Intensity (mAU)
521 nt ssRNA (1)
Retention time (min)
250
200
100
0
-50
0.0
5.0
10.0
15.0
20.0
23.1
Intensity (mAU)
521 nt ssRNA (2)
Retention time (min)
B
140
100
Intensity (mAU)
50
-20
2.0
5.0
10.0
15.0
20.0
23.1
Retention time (min)
C
686 bp dsRNA
D
220
150
Intensity (mAU)
100
50
-20
2.0
5.0
10.0
15.0
20.0
23.1
Retention time (min)
561 bp dsRNA
Supplementary Figure 1

## Slide 2
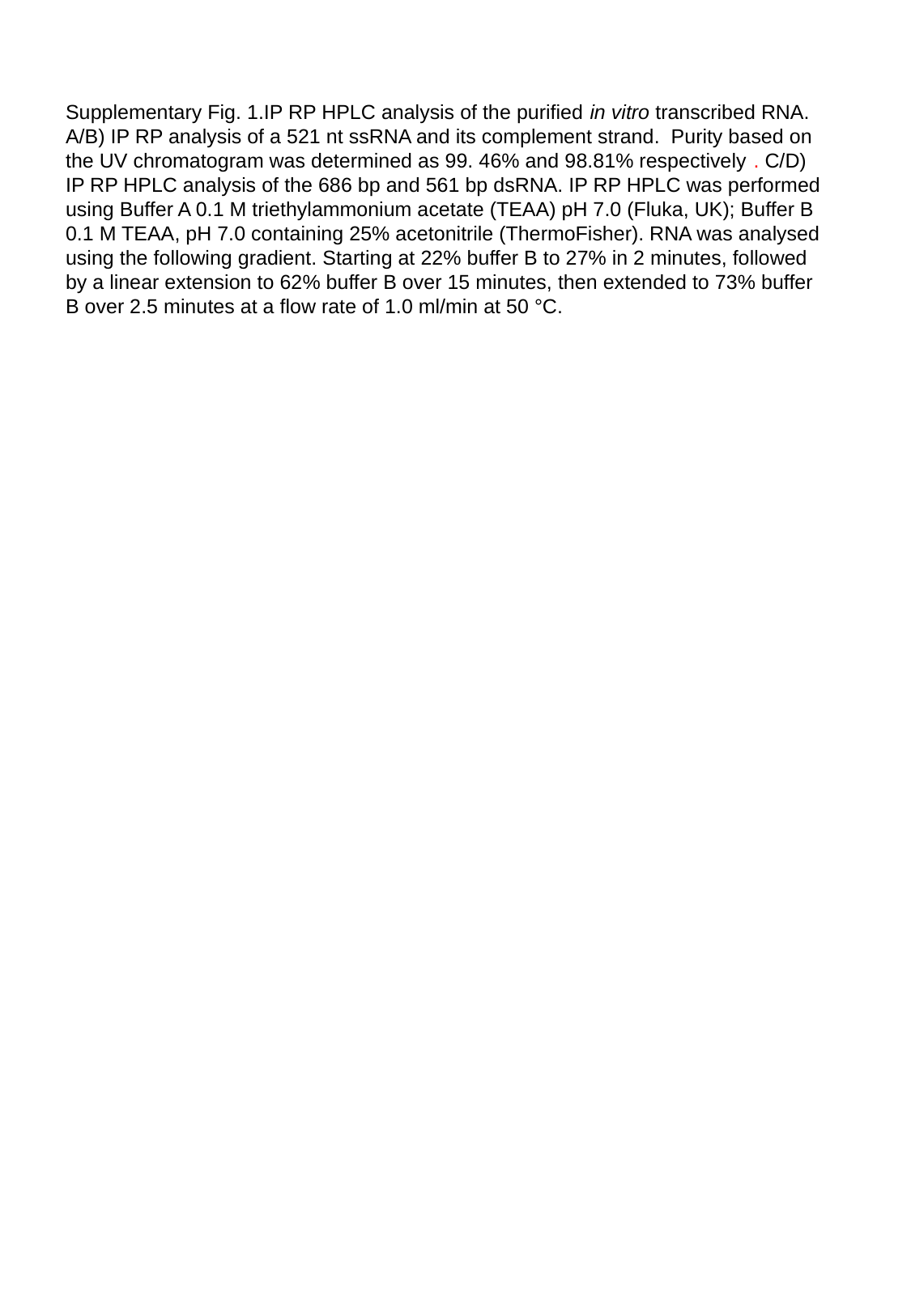

Supplementary Fig. 1.IP RP HPLC analysis of the purified in vitro transcribed RNA. A/B) IP RP analysis of a 521 nt ssRNA and its complement strand. Purity based on the UV chromatogram was determined as 99. 46% and 98.81% respectively . C/D) IP RP HPLC analysis of the 686 bp and 561 bp dsRNA. IP RP HPLC was performed using Buffer A 0.1 M triethylammonium acetate (TEAA) pH 7.0 (Fluka, UK); Buffer B 0.1 M TEAA, pH 7.0 containing 25% acetonitrile (ThermoFisher). RNA was analysed using the following gradient. Starting at 22% buffer B to 27% in 2 minutes, followed by a linear extension to 62% buffer B over 15 minutes, then extended to 73% buffer B over 2.5 minutes at a flow rate of 1.0 ml/min at 50 °C.

## Slide 3
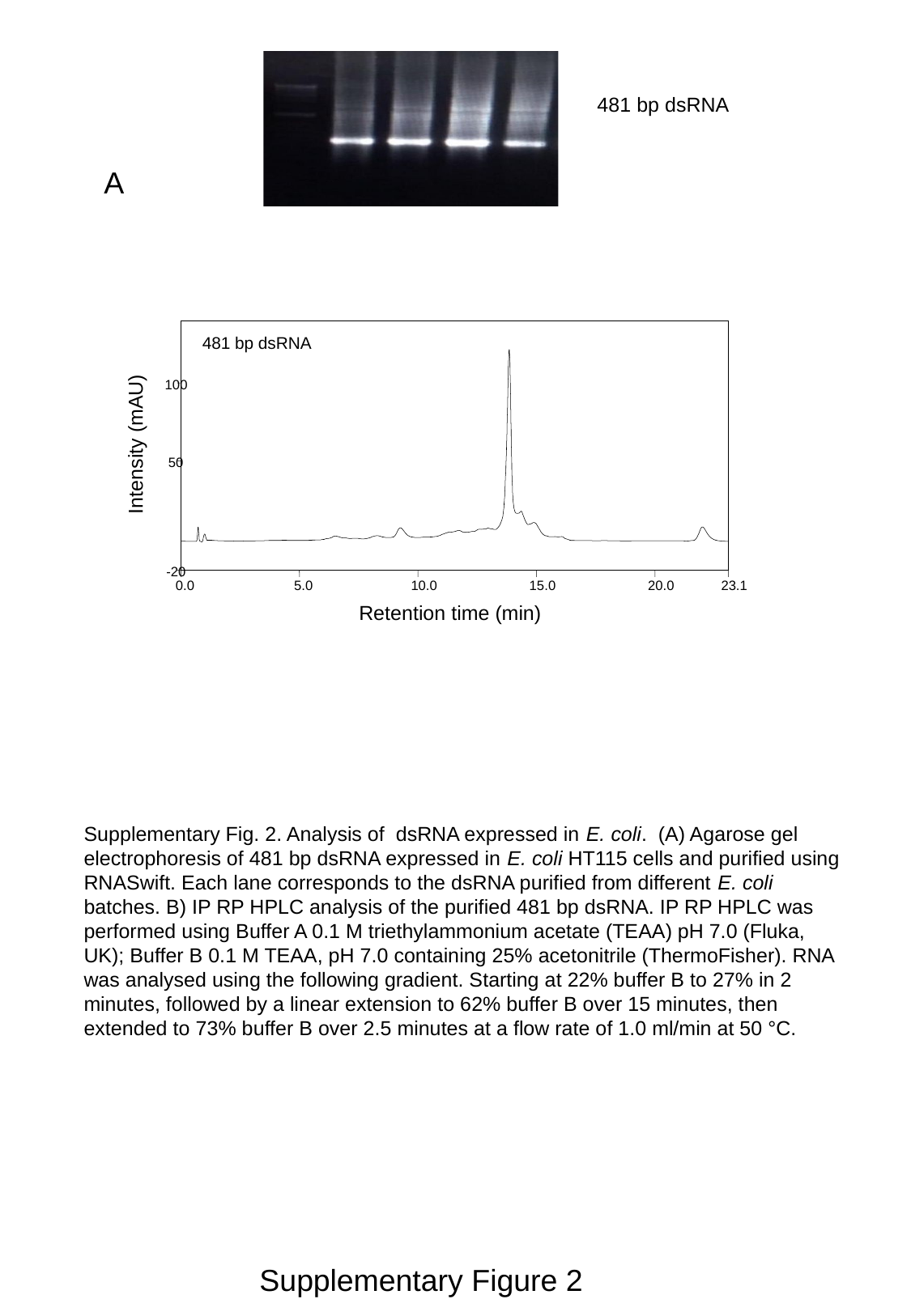

481 bp dsRNA
A
481 bp dsRNA
100
Intensity (mAU)
50
-20
0.0
5.0
10.0
15.0
20.0
23.1
Retention time (min)
Supplementary Fig. 2. Analysis of dsRNA expressed in E. coli. (A) Agarose gel electrophoresis of 481 bp dsRNA expressed in E. coli HT115 cells and purified using RNASwift. Each lane corresponds to the dsRNA purified from different E. coli batches. B) IP RP HPLC analysis of the purified 481 bp dsRNA. IP RP HPLC was performed using Buffer A 0.1 M triethylammonium acetate (TEAA) pH 7.0 (Fluka, UK); Buffer B 0.1 M TEAA, pH 7.0 containing 25% acetonitrile (ThermoFisher). RNA was analysed using the following gradient. Starting at 22% buffer B to 27% in 2 minutes, followed by a linear extension to 62% buffer B over 15 minutes, then extended to 73% buffer B over 2.5 minutes at a flow rate of 1.0 ml/min at 50 °C.
Supplementary Figure 2

## Slide 4
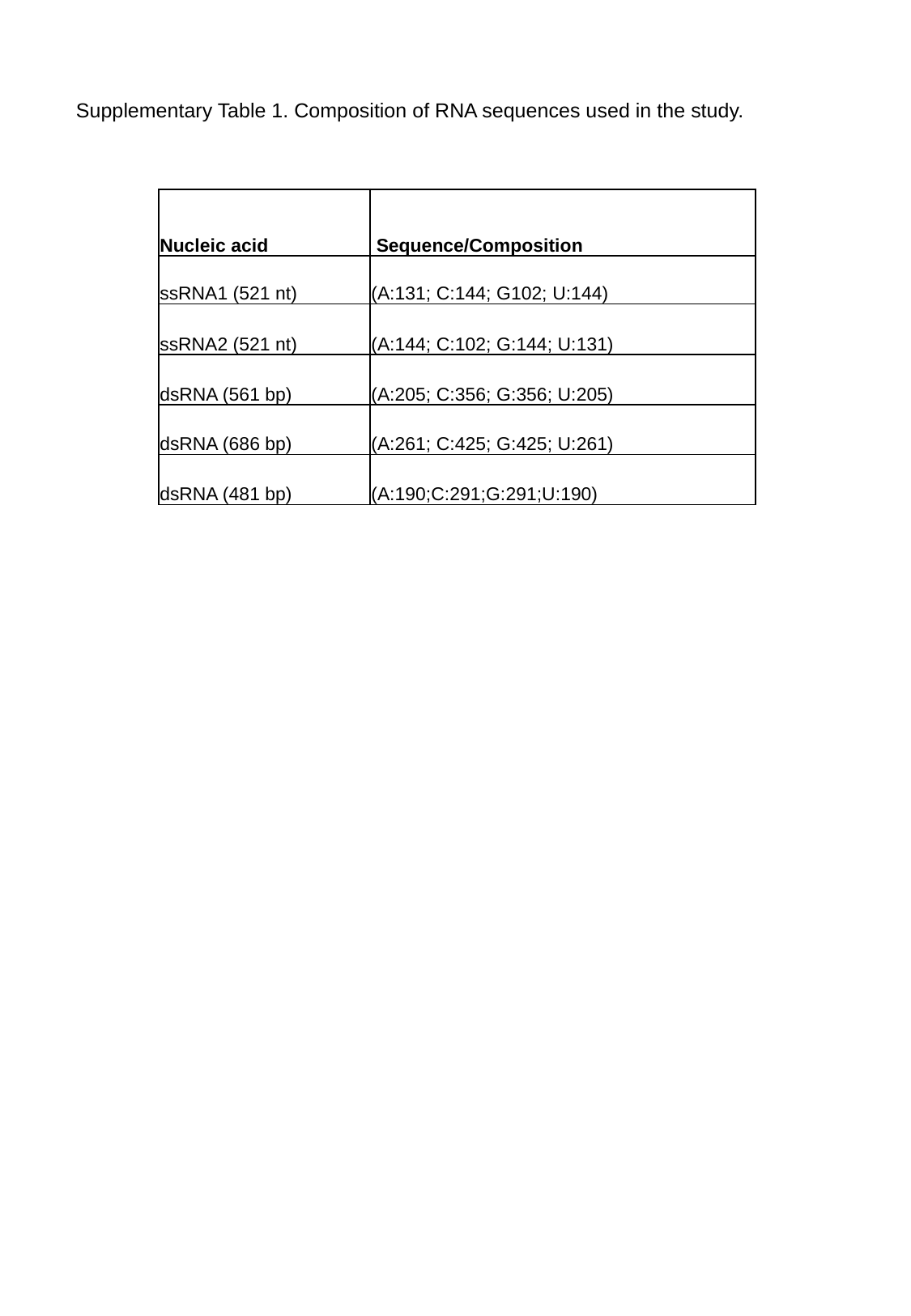

Supplementary Table 1. Composition of RNA sequences used in the study.
| Nucleic acid | Sequence/Composition |
| --- | --- |
| ssRNA1 (521 nt) | (A:131; C:144; G102; U:144) |
| ssRNA2 (521 nt) | (A:144; C:102; G:144; U:131) |
| dsRNA (561 bp) | (A:205; C:356; G:356; U:205) |
| dsRNA (686 bp) | (A:261; C:425; G:425; U:261) |
| dsRNA (481 bp) | (A:190;C:291;G:291;U:190) |
